# A Carboxyethylchitosan Gel Cross-Linked with Glutaraldehyde as a Candidate Carrier for Biomedical Applications

**DOI:** 10.3390/gels9090756

**Published:** 2023-09-16

**Authors:** Anastasia Korel, Alexander Samokhin, Ekaterina Zemlyakova, Alexander Pestov, Elena Blinova, Maxim Zelikman, Vadim Tkachenko, Viktoria Bets, Svetlana Kretien, Elena Arzhanova, Ekaterina Litvinova

**Affiliations:** 1 Faculty of Physical Engineering, Novosibirsk State Technical University, 630073 Novosibirsk, Russia; akorel@gmail.com (A.K.); blinovaelena-85@yandex.ru (E.B.); vish22@yandex.ru (V.B.); ssonovo64@inbox.ru (S.K.); e.arzhanova@g.nsu.ru (E.A.); dimkit@mail.ru (E.L.); 2 Institute of Organic Synthesis n.a. I. Ya. Postovsky UB RAS, 620137 Ekaterinburg, Russia; kottazem@mail.ru (E.Z.); pestov@ios.uran.ru (A.P.); 3 Institute of Solid State Chemistry and Mechanochemistry SB RAS, 630090 Novosibirsk, Russia; zelikman_mv@mail.ru; 4 Institute of Nuclear Physics SB RAS, 630090 Novosibirsk, Russia; vtkachen@mail.ru; 5 Novosibirsk Research Institute of Traumatology and Orthopedics, 630091 Novosibirsk, Russia

**Keywords:** carboxyethylchitosan, gel, photon correlation spectrophotometry, bacteriophages, acidic, alkaline, medium, gastrointestinal tract

## Abstract

To date, few publications describe CEC’s properties and possible applications—thus, further evaluation of these properties is a point of interest. The present in vitro model study aimed to evaluate a carboxyethylchitosan (CEC) gel with a degree of substitution of 1, cross-linked with glutaraldehyde at a polymer:aldehyde molar ratio of 10:1, as a potential carrier for delivering bacteriophages to various pH-fixed media (acidic, alkaline), and including gastrointestinal tract (GIT) variable medium. A quantitative analysis of bacteriophages released from the gel was performed using photon correlation spectrophotometry, and phage activity after emission into medium was evaluated using the spot test. The results showed that the CEC gel’s maximum swelling ratios were at a nearly neutral alkaline pH. Increasing temperature enhances the swelling ratio of the gel independent from pH, up to 1127% at 37 °C and alkaline pH. The UV and photon correlation spectrophotometry showed equal gel release kinetics in both fixed media with acidic (pH = 2.2) and alkaline (pH = 7.4) pH environments at 37 °C, with the maximum release within two hours. However, phage lytic activity in the spot test during this simulation was absent. At the same time, we obtained an opaque phage lytic activity in the alkaline pH-fixed medium for at least three hours. Phages released from the tested CEC gel in different pHs suggest that this gel could be used for applications that require fast release at the treatment site both in acidic and alkaline pH. Such treatment sites could be a wound or even soil with mild acidic or alkaline pH. However, such CEC gel is not suitable as a delivery system to the GIT because of possible transported acid-sensitive agent (such as phages) release and destruction already in the stomach.

## 1. Introduction

Significant progress in the organic chemistry of the last decade, along with emerging demand from various industries and medicine, has led to a large number of gels from materials of both natural and synthetic origin. Such materials, as well as their combinations, open up great prospects for their application in a wide range of tasks—from agriculture to biology and medicine [1,2,3,4,5].

Growing interest in phage therapy over the past decade has encountered a number of barriers, among which are applied tasks related to the delivery of phages to the treatment site. Phage delivery to wound surfaces or to the intestine requires protection from aggressive environmental factors to maintain the maximum possible phage titer and their lytic activity. Such protection is required to preserve phages’ therapeutic effect, as the optimal pH range for the functioning of phages lies within 6.0–8.0.

In the case of biomedical application, hydrogels from raw materials of natural origin are often more preferable, which is reflected in the numerous works on the creation of various implants, carriers for drug delivery, and scaffolds for tissue engineering [2,6,7,8]. One of these natural polymers is chitosan, known for its biocompatibility, lack of toxicity, sorption ability, and biodegradability due to the presence of amino, hydroxyl, and ether functional groups [2,9].

Among different chitosan derivatives, one could have a particular biomedical interest—carboxyethylchitosan (CEC). As a carboxyl derivative, CEC has a number of additional useful characteristics [10]: solubility in water in a wide pH range [11], better biodegradability [12], excellent antioxidant characteristics, significant antimutagenic activity [13], and better gelling ability [14] than chitosan. At the same time, few publications [15,16,17,18] describe CEC’s properties and possible applications—thus, further evaluation of these properties is a point of interest.

The presence of carboxyalkyl groups in the chitosan molecule enhances polymer affinity to the cell substrates [19,20]. In our previous study [21], CEC demonstrated one of the best cytotoxicity results in the human fibroblast cell culture and did not cause cell death. Based on these results, we decided to characterize CEC’s structure and swelling performance, along with its phage delivery properties, for its use as a potential carrier for delivering phages in an in vitro gastrointestinal tract (GIT) pH environment model simulation of a laboratory animal (mouse), in two pH-fixed medium (alkaline or acidic) variants, and as a candidate gel for biomedical application. Another aim was to focus on the quantitative results of the phage particles released using photon correlation spectroscopy, as a method for the count of high-precision small particles, and of a phage lytic activity evaluation using the spot test.

## 2. Results

### 2.1. Spectral and Morphological Characteristics of CEC Gel

**Figure 1:**
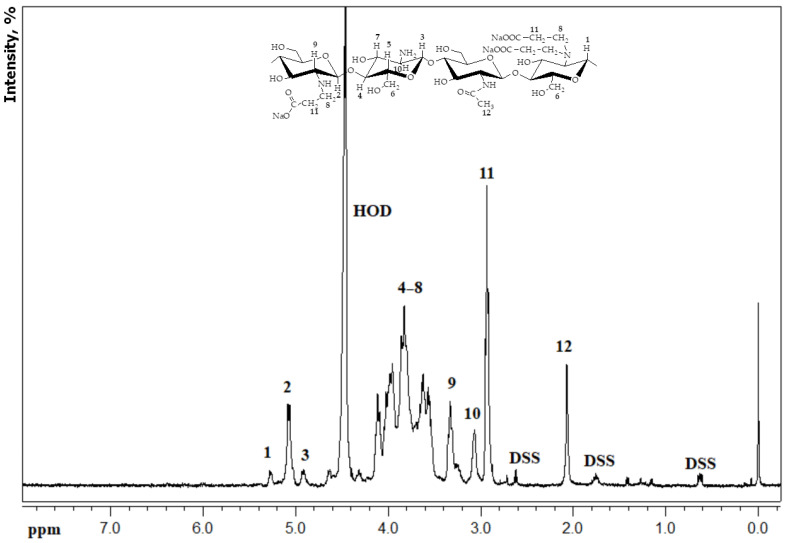
A 400 MHz ^1^H NMR spectrum of N-(2-carboxyethyl)chitosan with degrees of modification 1.0 (D_2_O/DCl) (DSS—2,2-dimethyl-2-silapentane-5-sulfonate sodium salt was used as an internal ^1^H NMR standard; ppm—parts per million). Numbers above the peaks on the spectrum graph and in the structure formula are hydrogen atoms, corresponding to each other.

CEC was synthesized using polymer transformations in the gel state of chitosan in the addition reaction to acrylic acid [22]. CEC synthesis was confirmed with elemental analysis, FT-IR spectroscopy (Figure S1), and potentiometric titration. A selective addition of the amine groups with degrees of modification 1.0 was confirmed with ^1^H NMR spectroscopy. The spectrogram in Figure 1 shows signals of the hydrogen atoms in the main polymer chain (2.07 (CH_3_), 3.07 (H-2 GlcNH_2_), 3.44–4.10 (H-2,3,4,5,6), 4.63 (H-1 GlcNHAc), 4.92 (H-1 GlcNH_2_) ppm) and new carboxyethyl groups (3.35 (NHCH_2_CH_2_), 3.33 (H-2 GlcNHCH_2_) ppm). Signals at 4.92, 5.06, and 5.25 ppm correspond to the H-1 atom of glucosamine units containing primary, secondary, and tertiary amine, respectively, which confirms the N-functionalization of the chitosan.

**Figure 2:**
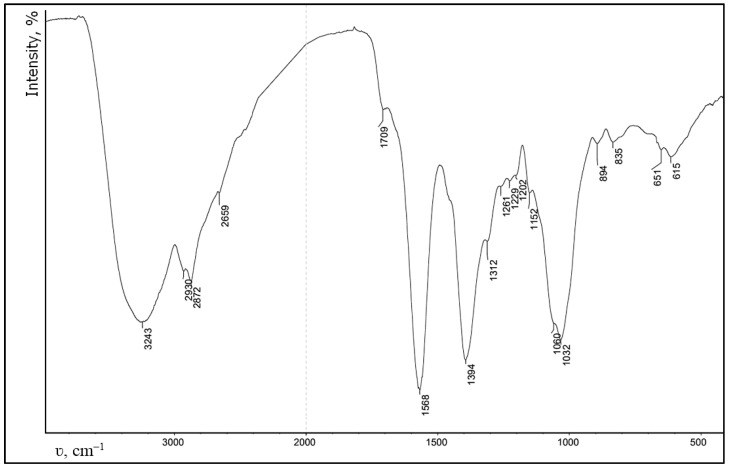
FT-IR spectrum of N-(2-carboxyethyl)chitosan cross-linked with glutaraldehyde.

CEC gelation was performed with glutaraldehyde as a cross-linker [22]. Results of the FT-IR spectra (Figure 2) of the product demonstrate that aside from absorption bands characteristic for the CEC molecule at 3243 (O−H, N−H), 2872 (C−H), 1709 (COOH), 1568, 1394 (COO^−^), and 1060, 1032 (C−O, C−C) cm^−1^, a new very weak band emerged at 1641 cm^−1^ that is characteristic for valence vibrations of the C=N imine bond, which was produced during CEC primary amine groups and glutaraldehyde interaction.

**Figure 3:**
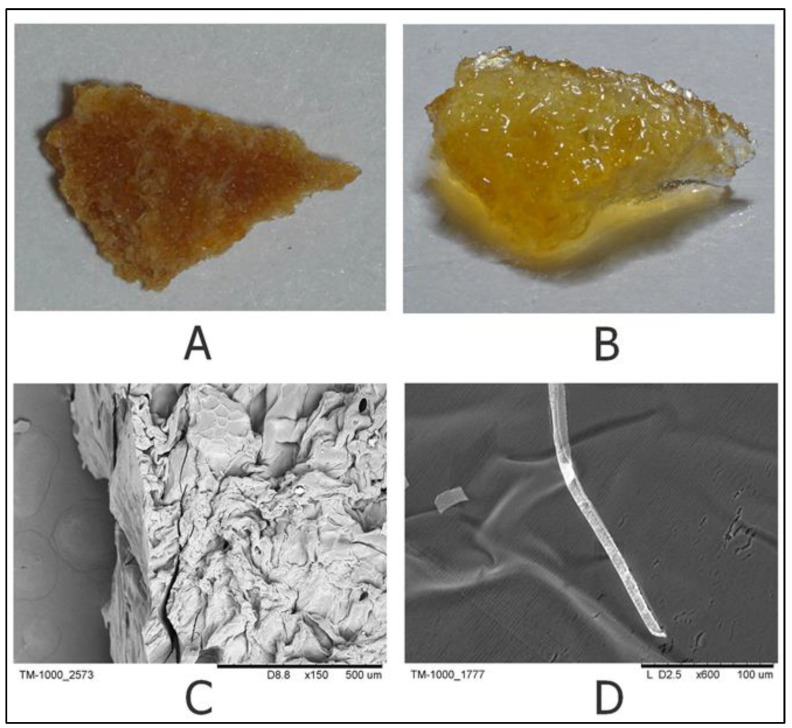
Macroscopic images and SEM of CEC gel. Macroscopic image of (**A**) dry gel and (**B**) swollen gel. SEM of the surface of CEC gel in a (**C**) dry and (**D**) swollen state, with 600× magnification.

The appearance of the tested gel in dry form and after swelling (macroscopic images and scanning electron microscopy (SEM)) is shown in Figure 3. A dry gel sample at ×150 magnification on SEM images has a developed surface with multiple large cavities (Figure 3A,B). The swollen gel has an almost smooth surface over its entire area (Figure 3C,D), which is due to an increase in the size of the chitosan matrix and is associated with the size increase and stretching of the surface.

**Figure 4:**
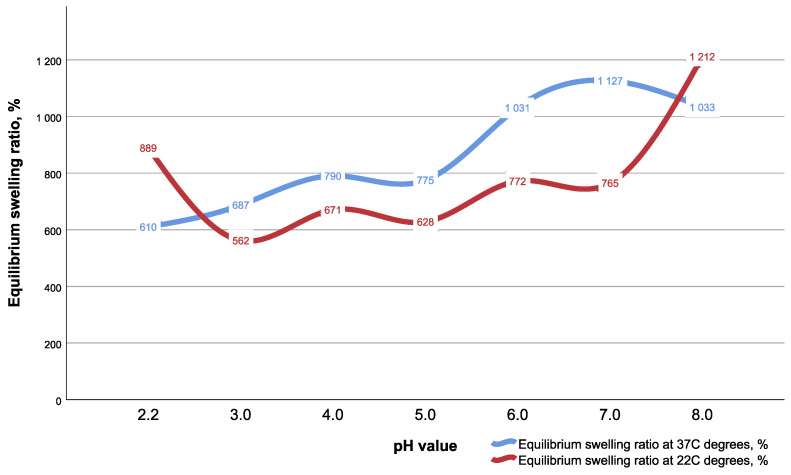
Swelling performance of CEC gel at two temperature values and range pH from 2.2 to 8.0.

CEC gel shows pH- and temperature-dependent swelling performance, with maximum swelling ratios achieved at neutral and alkaline pH values (Figure 4). An increase in temperature leads to a rise in the swelling ratio for most pHs. The swelling ratio increase started from pH = 3.0 and was maintained up to normal pH values, and even for alkaline pH where the swelling performance was 1033%. However, the maximum swelling of gel was 1212% in alkaline pH at 22 °C.

The observed dependence of an increase in the degree of swelling with an increase in pH (Figure 4) is entirely due to the amino acid nature of CEC. In an acidic environment, CEC molecules are in protonated form [11]. With increasing pH, the degree of dissociation of carboxyl groups increases, which leads to an increase in the number of more polar carboxylate groups. In addition, the degree of hydration, i.e., the number of water molecules per mole of CEC molecules, increases with an increase in pH, which is reflected in the change in the degree of swelling.

### 2.2. Release Rate of CEC Gel under Various pHs

The release rate for the gel, loaded with bacteriophages and dye, incubated with acidic pH-fixed medium (equal to 2.2) or alkaline pH-fixed medium (7.4) at 37 °C, showed maximum release capability in the acidic pH followed with a gradient decrease in the medium until the fourth hour.

**Figure 5:**
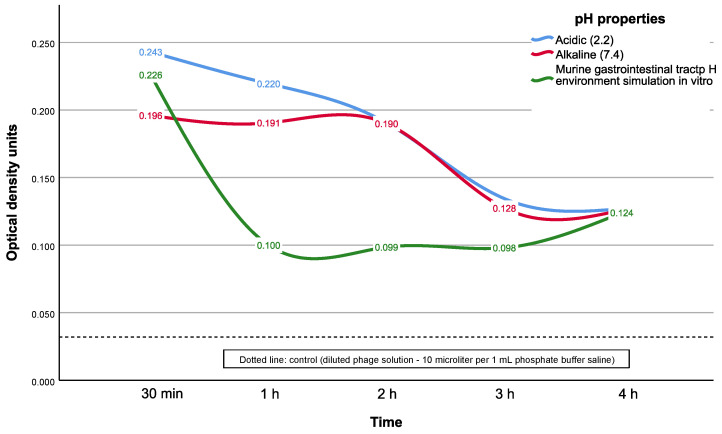
Dye release kinetics of the CEC gel for different pH media at 37 °C. Note: data presented as median values.

We revealed decreasing ODUs of dye for 1.5 times in alkaline pH medium from 0.196 [0.194; 0.198] to 0.128 [0.123; 0.130] ODUs and for 1.9 times in the acidic pH from 0.243 [0.237; 0.249] to 0.135 [0.123; 0.136] ODUs from 30 min up to the third hour (*p* = 0.500 in both cases, with Bonferroni correction for multiple comparisons) (Figure 5).

A similar decrease in ODUs of dye recorded for the release rate of the CEC gel incubated with a variable pH switched from acidic pH 2.2 to alkaline pH 7.4 at 37 °C. The ODUs of dye during release from the gel to the medium almost halved, from 0.226 [0.179; 0.276] to 0.100 [0.093; 0.106] ODUs (*p* = 0.001) (Figure 5).

This suggests that under such conditions the dye release from the gel in the acidic medium occurs quickly. By the time the gel is transferred to the alkaline medium in 30 min, the dye has already been released.

**Figure 6:**
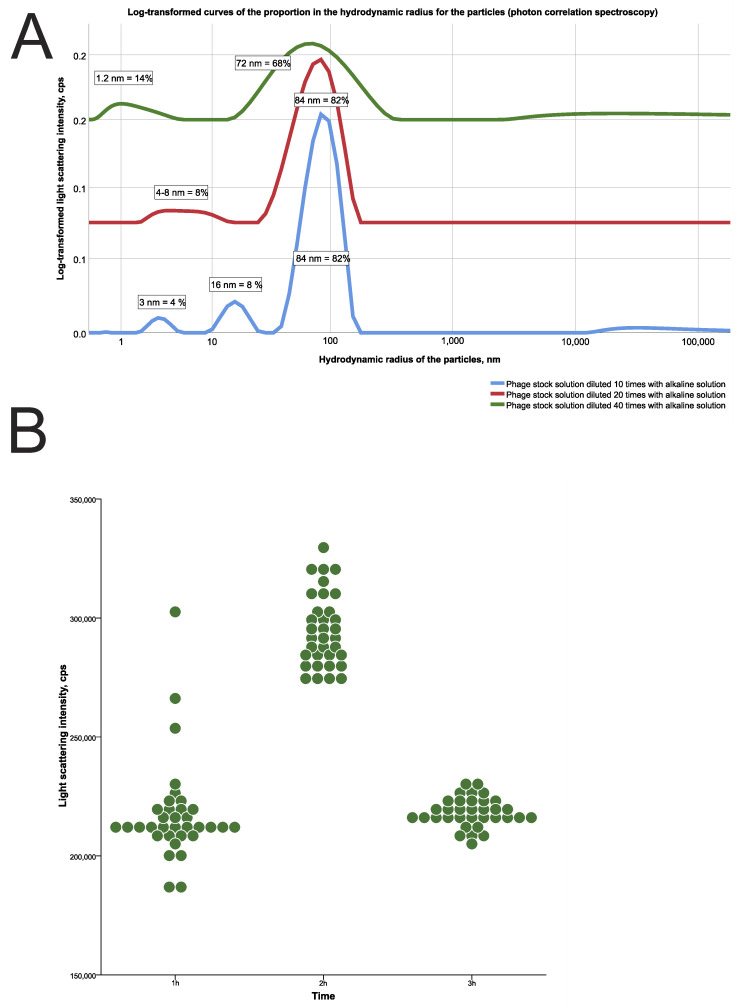
Results of the photon correlation spectrophotometry of phages released from CEC gel in an in vitro pH environment GIT simulation. (**A**) Particle size of phages diluted 10, 20, and 40 times. (**B**) Count of undiluted phages released from CEC gel at the first, second, and third hours assayed by light-scattering intensity. Note: cps—counts per second.

Most of the bacteriophages released from CEC gel assayed by photon correlation spectrophotometry in variable pH as in the GIT had a particle size of about 80–100 nm (Figure 6A). The maximum intensity of the light scattering by particles was recorded on the second hour (Figure 6B). This indirectly demonstrates that the maximum concentration of phages in alkaline pH was reached after two hours. Changes in the rate of phage release after two hours (291,566.6 [282,817.19; 303,594.40] cps (counts per second)) were statistically 1.4 times higher compared with those after one (213,148.4 [209,954.09; 219,607.48] cps) and three (217,797.7 [215,545.83; 222,730.42] cps) hours (*p* < 0.00001, Friedman test).

The detected phage size corresponds to our calibration measurements: 82% of all detectable particles in stock solution of bacteriophages had a size of 84 nm (Figure 6A). This is the average size of bacteriophages described in the literature [23]. Results also show that the maximum particles count recorded with photon correlation spectrophotometry was detected at the second hour, while the highest ODU values by UV spectrometry, as a dye release indicator, were highest at the first hour (Figure 5). We assume that the diffuse release of the dye from the gel is faster than the release of phages due to the acidic nature of the dye used that decreases the sorption for CEC while the phage stock solution is near pH neutral values. Another factor could be the higher diffusion coefficient of dye in which molecules should be smaller than phages.

### 2.3. Lytic Activity of Bacteriophages Released from CEC Gel

**Figure 7:**
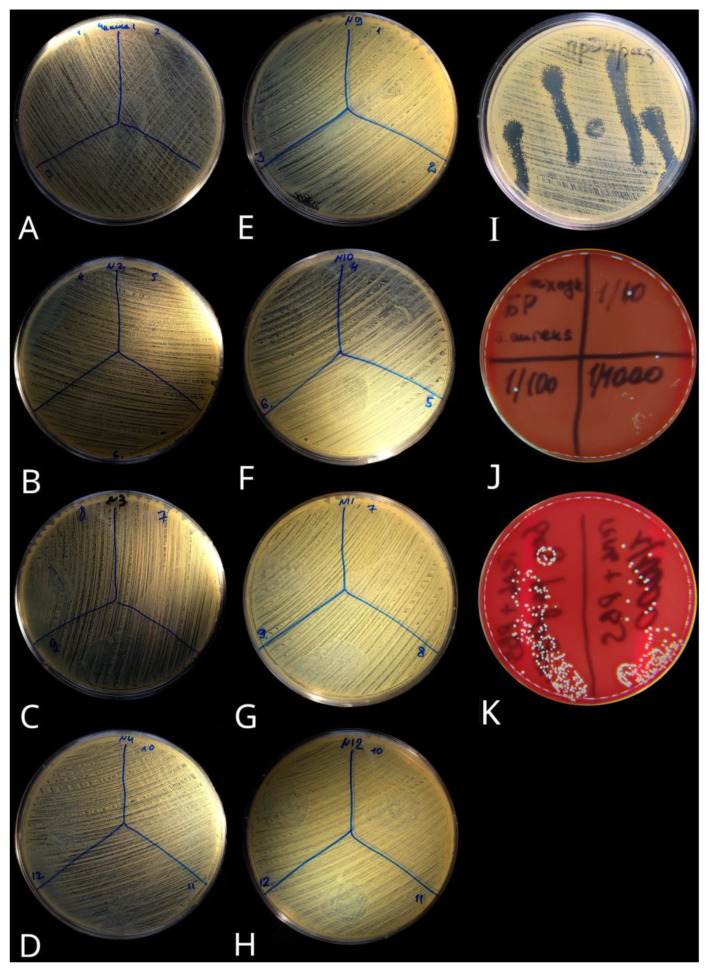
Lytic activity of phages (undiluted) released from CEC gel in alkaline (7.4) and variable pH simulated GIT. (**A**–**D**) Lytic activity of phages released from CEC gel in 30 min, 1 h, 2 h, and 3 h exposure by variable pH that simulated the GIT, respectively (performed in triplicate); (**E**–**H**) Lytic activity of phages released from CEC gel in 30 min, 1 h, 2 h, and 3 h of exposure in alkaline (pH 7.4) fixed medium, respectively (performed in triplicate); (**I**) Lytic activity of phages in stock solution (control); (**J**) Bacterial growth test with CEC gel loaded with phages and released in a 0.85% sodium chloride solution with suspension of *S. aureus* test strain (plate divided into four sections: undiluted suspension and diluted by 10, 100, and 1000 times); (**K**) Bacterial growth test with CEC gel loaded with phosphate-buffered saline (phageless) and released in a 0.85% sodium chloride solution with suspension of *S. aureus* test strain (undiluted bacterial test strain on the left and 1000 times dilution on the right section of the plate).

Simulation of the in vitro phages releasing from CEC gel passing through the pHs of the GIT followed by microbiological tests, performed in triplicate, did not show lytic activity against *S. aureus* in the first hour and later (Figure 7A–D). The opaque lysis of bacterial lawn with *S. aureus* in the spot test that we had found at 30 min of the gel exposure in acid had acidic but not phage genesis against, as bacteriophages and bacteria are sensitive to an aggressive acidic pH environment and are destroyed at pH = 2.2 (Figure 7A–D). In addition, this was a reason not to test phages with the acidic pH-fixed medium in our study.

At the same time, we observed an opaque lytic activity in the alkaline pH (7.4) fixed medium during three hours of exposure of undiluted phages, which may indicate a phage emission from the CEC gel (Figure 7E–H). Phage activity in identical pH = 7.4 was confirmed also in the control (Figure 7I). It should be mentioned that dilution by 2 and 4 times in alkaline pH (7.4) fixed medium preserves the opaque lytic activity of phages, but higher dilutions of phages reduce the lytic activity instead. The dilution of phages when released from the CEC gel at the second hour was more than 10 times, so the lytic effect was weakly expressed. Therefore, releasing phages from CEC gel and their dilution in medium negatively affects the titer required for their lytic activity.

An additional in vitro test performed to confirm phage viability in their typical pH usage conditions showed that phages being released from the gel almost completely eliminated the bacterial test strain in the bacterial suspension—only a few colonies were visible after subsequent 100 μL aliquot seeding to the Petri dishes with blood agar medium to detect possible bacterial test strain growth (Figure 7J). On the contrary, the phageless control (CEC gel loaded with phosphate-buffered saline only) had numerous colonies on all plate sections (Figure 7K), because CEC gel has no antibacterial properties itself. Thus, the tested CEC gel demonstrates an absence of phages protection during in vitro simulation of the phage-loaded gel passage through the acidic pH of the GIT but may be suitable in other biomedical applications with different pH conditions.

## 3. Discussion

To date, various transport carriers for phages delivery to the treatment site have been developed and presented in the available literature; in particular, carriers based on alginate, polyethylene glycol, chitosan nanoparticles, liposomes, and a number of other carriers have been described [24,25,26]. One of the primary tasks for any phage carrier is an analysis of the bacteriophages release, as their classic titration takes a certain time, which can be critical for the mass screening of candidate carriers. At the same time, the candidate selection process is complicated by the fact that it is necessary to take into account that carrier properties could be altered by the sterilization process [27], and sometimes in a negative way.

The experimental setup we created for the physical separation of the gel sample and the agent being released from it into the model environment by diffusion (in this case, bacteriophages) in the original version was planned as filter-equipped. The filtration was to be performed through commercially available cell filters with a pore diameter of 70 μm for the physical division of the container on two compartments. The first compartment was where the gel samples were placed, and the second one was where the transported agent should be released to prevent the possible entry of tested carrier microfragments into the buffer medium and subsequent plate reading errors during UV spectrophotometry measurements.

Nevertheless, we have noted that the tested gel sample does not undergo fragmentation and does not form suspensions of colloidal particles in the buffer medium when being exposed to buffer media with different pH values; thus, UV spectrophotometry measurements remain unaffected. This makes it possible to discard from the polymer cell filter usage for the container compartment division in such an experimental setup to filter out possible carrier degradation microproducts. In turn, such simplification makes it possible to reduce the required amount of the buffer medium where agent release from the carrier occurs. However, we believe that for each new tested gel sample (with a different chemical composition) this parameter will need to be evaluated each time to prevent possible UV spectrophotometry measurement errors.

The relevant experimental setups found in the literature for in vitro GIT simulation of the impact of an acidic environment of the stomach and enzymes on chitosan nanoparticles, preloaded with bacteriophages [26] or heparin [28], had their own specifics. The first model [26] was built as a single container with the requirement of the aliquoted buffer medium volume to be replenished with the same new equal buffer medium volume, with simultaneous usage of one of the two variants for simulating the environment of the stomach (with and without pepsin as an enzyme component of the model). The second model [28] was the prototype of the first one, equipped with an extended enzyme set, pepsin for an acidic environment and pancreatin for an alkaline environment, although it was not characterized in detail by its authors.

However, in our own model, we did not replenish the medium during the experiment because, in our opinion, the replenishing of the aliquoted medium volume after each aliquot sampling will affect the light-scattering results. This could happen when performing both UV and photon correlation spectrophotometry, thereby the results could be distorted.

Our results of the tested CEC gel release kinetics analysis have demonstrated a very fast dye release rate during the first two hours of incubation in fixed medium with alkaline or acidic pH. In the case of modeling the pH environment of the murine GIT, the protonation of chitosan amino groups with a change in the model environment from acidic to alkaline probably occurs very quickly in our experimental setup. Such conditions could induce chitosan degradation and trigger the rapid release of the dye (but phages could be damaged in this way due to contact with acid) into the buffer medium. However, in the case of using a fixed medium pH buffer, the release time interval was longer, with the relatively stable ODU release values according to the results of UV spectrophotometry during the first two hours of the experiment.

That such a rapid release from the carrier could be a result of the lytic activity of phages, being released from the carrier, was not confirmed by the bacteriological method in the spot test. Indeed, the phage titer during in vitro GIT simulation was insufficient to demonstrate lytic activity against the target *S. aureus* test strain because most of the phages were destroyed by acid. Despite the released phage particles demonstrating a detectable titer of numerous light-scattering particles with a size of about 100 nm by the photon correlation spectrophotometry, it should be noted that most of those particles could be acid-inactivated phages, which have no lytic activity.

At the same time, we observed opaque phage lytic activity in the alkaline pH-fixed medium, which may indicate a phage emission with near effective (lytic) titer from the carrier in a slightly alkaline or normal pH environment. Based on these results, one could suggest that an effective (i.e., therapeutic) phage titer with the tested CEC gel could be obtained in the alkaline pH environment with around normal pH values. In addition, such a titer could be maintained for at least three hours, in the case of the high and sufficient initial stock phage titer before gel swelling. Phage activity in almost the same pH conditions was also confirmed in the control (pH = 7.2 for the Mueller–Hinton agar; the same agar was used for the alkaline pH aliquots), which proves our suggestion. However, it should be taken into account that phage viability mostly relies on the optimal pH conditions of the phage strain itself. This was also proven in our additional in vitro experiment with the same phage strain and batch, which confirmed phage viability in the optimal pH (near neutral) usage conditions and demonstrated effective bacterial test strain elimination after phage release from the CEC gel.

Thus, the tested CEC gel could be used in the future as a carrier system for applications that require the fast release of the transported agent, both in acidic and alkaline fixed pH environments, i.e., wound healing or even acidic pH soils due to mild acidic pH conditions than in our study. However, it is not suitable for usage as a carrier for GIT delivery because of possible massive transported agent (such as phages) release and destruction already in the stomach with its aggressive acidic environment.

At the same time, this experiment showed the possibility of bacteriophages release detection from the gel carrier using photon correlation spectrophotometry. We did not encapsulate bacteriophages in a gel carrier but loaded the carrier by the natural swelling of the chitosan matrix when the gel sample was immersed in a phage stock solution. Thus, only the phages could be present, detected as numerous particles released from the carrier in the buffer medium.

The following factors are the limitations of our experiment: The first is the absence of an enzyme in the in vitro GIT simulation buffer media, as the addition of such an agent as pepsin would increase the rate of chitosan degradation and at the same time could accelerate the release rate. However, despite the fact that the pepsin presence in the model medium could affect the concentration of bacteriophages during the experiment, there is evidence [29] that chitosan carrier usage could effectively protect bacteriophages in an acidic pH environment, including possible pepsin action. Therefore, the next step in such an in vitro GIT simulation experimental setup could be the addition of pepsin and enzyme components into acidic and alkaline buffer media for further tested gel evaluation.

The second limitation is the phage strain used for the experiment because of vulnerability to the aggressive acidic pH conditions (pH = 2.2). However, the same strain has demonstrated effective bacterial lysis in fixed-pH medium (near-neutral values) and probably could demonstrate effective bacterial lysis in a mild acidic pH range (5.5 and above), which was not tested in our experiment.

## 4. Conclusions

The CEC gel being tested showed nearly equal gel release kinetics in both acidic and alkaline pH environments, with the maximum release within two hours followed by smooth kinetics decrease after that.

There was no phage lytic activity in the spot test during the in vitro GIT environment simulation due to phages that were destroyed being massively and rapidly released in the strong acidic pH environment, despite the detected phage particles of 80–100 nm released within two hours in the buffer medium, with the maximum in the second hour.

Such release kinetics in different pH environment variants suggest that this carrier could be used in the future for applications that require the fast release of the transported agent at the treatment site, both in acidic and alkaline fixed pH environments. For example, it could be used in wound healing or even acidic pH soils due to their mild acidic pH conditions than in our study. However, it is not suitable for usage as a carrier for GIT delivery because of possible massive transported acid-sensitive agent (such as phages) release and destruction already in the stomach with its aggressive acidic environment.

## 5. Materials and Methods

As a test sample, we used a CEC gel with the degree of substitution of 1, cross-linked with glutaraldehyde at a polymer:aldehyde molar ratio of 10:1, synthesized using a previously developed method [22]. The composition and structure of the synthesized gel were confirmed by elemental analysis using “CHN PE 2400” (Perkin Elmer, Waltham, MA, USA) and FT-IR spectroscopy using a “Spectrum Two” spectrometer (Perkin Elmer, Waltham, MA, USA).

Before the experiment the gel was sterilized according to the previously selected method, electron beam sterilization with an absorbed dose of 25 kGy [27], and the experiment was carried out using sterile samples only.

### 5.1. Swelling Analysis of CEC Gel

The swelling properties were measured using the gravimetric method, with the pH in the range of 2.2–8.0 (with 1.0 increment between pH values) and two temperature modes, at room temperature and at 37 °C, to simulate live system thermal conditions. A swelling ratio (SR) and equilibrium swelling ratio (ESR) were calculated by the following formulas:

SR (%) = (mt − m_0_)/m_0_ × 100% (1)

ESR (%) = (m∞ − m_0_)/m_0_ × 100% (2)

where m_0_ is the mass of dry gel, mt is the mass of swollen gel at time t, and m∞ is the mass of the gel at equilibrium swelling.

In brief, to measure SR and ESR, a completely dry CEC gel of known mass (m_0_) was immersed for a definite time period in appropriate buffer solution and after time t, the sample was taken out of the solution and quickly wiped with filter paper and weighed (mt and m∞). Only ESR values are presented in the study results.

### 5.2. Scanning Electron Microscopy

Scanning electron microscopy of dry and swollen samples was performed with a TM-1000 microscope (Hitachi, Japan, Tokyo) after metallization of the samples with a gold layer 20 nm thick using a JFC-1600 (JEOL, Japan, Tokyo) at a pressure of no more than 8 Pa for 40 s.

### 5.3. Design of Experimental Setup

There was a pH equal to 2.2 for the stomach pH environment simulation. There were two pH values, equal to 7.0 and 7.4, for the intestine pH simulation, assuming the intestine pH range of normal to slightly alkaline [30]. The corresponding pH buffer solutions were prepared based on the recipe according to Mcilvaine T.C. [31].

**Figure 8:**
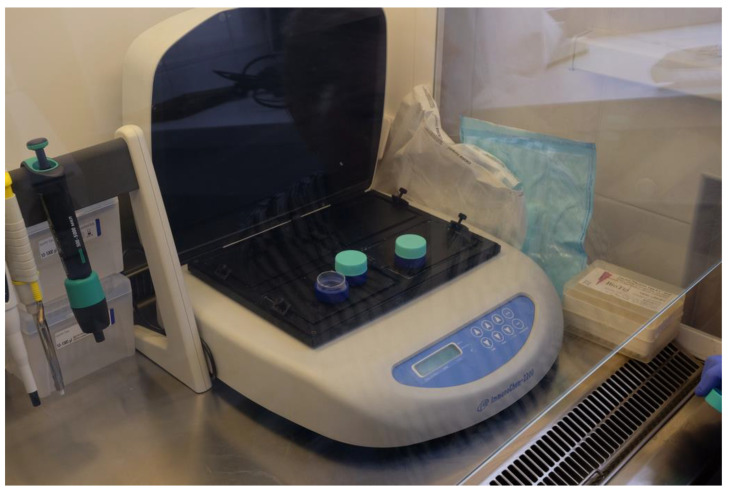
Experimental setup using a thermostatic shaker-incubator.

The hardware experimental setup (Figure 8) was based on the usage of plate shaker-incubator ImmunoChem-2200 (High Technology, North Attleboro, MA, USA) to maintain a liquid flow around the gel sample and the required temperature (37 °C). All gel samples were placed into separate small plastic containers with a volume of 5 mL, made from custom cut 50 mL polypropylene tube (falcon type) with a 70 μm cell porous filter (TPP, Switzerland, Trasadingen) installed to physically separate the container into two sections, lower and upper, to prevent gel fragment migration to the lower section and thus to avoid spectrometry artifacts.

Before the experiment, all prepared 5 mL containers were filled with one of three selected pH buffer solutions for the entire available internal volume, including the filter, and after that a phage-loaded gel was placed in the upper part of the container.

To detect the release from the gels using UV spectrometry, dye was added to the medium (acridone acetic acid in the form of a commercially available preparation of meglumine acridone acetate in 125 mg/mL ampoules, Polisan, St. Petersburg, Russia). We used this dye to visualize the release from the gel. Optical density of the acridone acetic acid was from 340 to 450 nm and the maximum absorption of that was 390 nm [32].

Gels were loaded with bacteriophages and dye (meglumine acridone acetate) using the next method: the solution, containing 0.5 mL solution of staphylococcal phage (sterile purified filtrate of phagolysates of *Staphylococcus* spp. with a titer at least 10^5^ PFU/mL, batch H265, production date May 2021, Microgen, Moscow, Russia) and 5.0 μL of meglumine acridone acetate, was incubated with a gel carrier during two hours at room temperature. This solution concentration was titrated to achieve the spectrophotometer lower detection threshold of optical density values for this dye. After loading the gels with a solution containing bacteriophages and dye, the gel samples were washed once in a 1× phosphate-buffered saline solution and placed in the 5 mL containers (described above), filled with an appropriate pH buffer.

The gel samples were then divided into **two study groups** to evaluate the gel release properties in different pHs:

The **first group** (n = 6) included gel for the assay of the release rate of the bacteriophages and dye acridone acetic acid, under two fixed medium pHs (alkaline pH 7.4 or acidic pH 2.2) at 37 °C with constant stirring for 4 h. Every 60 min, aliquots of 200 μL sample of medium from all containers were dropped into black 96-well plates with a translucent bottom for optical density analysis on a TriStar LB 941 spectrophotometer (Berthold Technologies, Germany) at 355 nm.

The **second group** (n = 6) included gel for the assay of the release rate of the bacteriophages and dye acridone acetic acid under pH that simulated the murine gastrointestinal tract (GIT), as the laboratory animal. The time intervals for the pH environment simulation was chosen as 30 min for acidic pH (2.2) medium and then switching to four hours for alkaline pH (7.4) medium, both at 37 °C. Every 15 min, aliquots of 200 μL sample of medium from all containers were dropped into 96-well plates for optical density analysis on a TriStar LB 941 spectrophotometer (Berthold Technologies, Germany, Bad Wildbad) at 355 nm.

To evaluate the ***lytic activity of phages released from the gels and quantify their absolute number***, the experiment was carried out under sterile conditions. The experimental setup described above (a shaker-incubator and containers with buffer solutions) was placed in a “Mars 1200” (Scanlaf, Vassingerod, Denmark) laminar flow and all manipulations were carried out within a laminar flow.

For this experiment, only the phage solution was loaded into gel samples for 2 h at room temperature. The absolute phage quantity was evaluated using a photon correlation spectrometer “Photocor Compact-Z” (“Photocor”, Moscow, Russia) after calibration of the device. The setting of this experiment with the gel was carried out according to the algorithm described in the paragraph about study groups above. To determine the releasing number of phage particles, every 60 min 200 μL aliquots of medium from all containers were transferred into sterile glass tubes for spectrometry.

### 5.4. Lytic Activity of the Bacteriophages

The lytic activity of the bacteriophages released from the gel was evaluated by the microbiological method (spot test for bacterial lawn lysis) on Petri dishes using Muller–Hinton agar. For this, a suspension of *S. aureus* test strain (ATCC 29213) dissolved in 0.85% sodium chloride with a density of 0.5 c.f.u. turbidity standard according to McFarland. Next, bacteria were added onto the selective medium in the Petri dishes to cultivate a bacterial lawn. Then, all aliquots from bacteriophages were dropped into Petri dishes with bacterial lawn in sterile conditions. The obtained lysis zones following overnight incubation at 37 °C were categorized as proposed by [33]: confluent lysis, semi-confluent lysis, opaque lysis, separate plaques, and no activity.

Two groups of test samples were prepared for this experiment:

Group 1: The bacteriophages count not absorbed by the gel during gel swelling was evaluated by taking 100 µL aliquots from the stock solution in which the gels reached equilibrium swelling during absorption. Next, these aliquots were added to Petri dishes to determine the bacteriophage lytic activity of the solution that was not absorbed into the gel.

Group 2: To determine the lytic activity of bacteriophages during the in vitro simulation of the GIT according to the algorithm described in the paragraph about study groups above. Both the gels themselves and the aliquots of the alkaline medium were taken from the containers in a volume of 1 mL. Then, aliquots were placed into the Petri dishes with bacterial lawn. Then the Petri dishes were incubated at 37 °C for 24 h, after which the phage lytic activity was assessed by the evaluation of bacterial lawn lysis zones.

For these tests two additional groups were used as controls:

Group 3: An undiluted 100 µL stock solution of staphylococcal bacteriophages (sterile purified filtrate of phagolysates of Staphylococcus spp. with a titer of at least 10^5^ PFU/mL, batch H265, production date May 2021, Microgen, Moscow, Russia).

Group 4: Stock solution of bacteriophages serially diluted 2, 4, 8, and 10 times with Dulbecco’s phosphate-buffered saline (with the selection of 100 μL aliquots for each Petri dish).

The phage viability was tested in a separate experiment. To do this, gel samples were placed in an undiluted stock solution of staphylococcal bacteriophages (the same phage stock batch H265 as described above) in which the gels reached equilibrium swelling during absorption. After that phage-loaded gels were washed with phosphate-buffered saline (PBS) and moved to test tubes with four variants of suspension of the *S. aureus* test strain (ATCC 29213) dissolved in 0.85% sodium chloride with a density of 0.5 c.f.u. turbidity standard according to McFarland. These variants were an undiluted suspension and diluted 10, 100, and 1000 times (performed in triplicate); all were incubated during three hours at 37 °C to allow phage emission in the tube’s liquid medium.

Then, gel samples were evacuated from the test tubes and test tube incubation continued in the same conditions up to 24 h, followed by 100 μL aliquots sampling of the liquid medium to evaluate phage viability. All aliquots were placed into the Petri dishes with blood agar medium to detect the possible growth of the *S. aureus* test strain and incubated in the “Incucell 707” (BMT, Brno-Zábrdovice, Czech Republic) for 24 h at 35 °C, followed by bacterial growth visual evaluation. As a control, PBS-loaded (without phages) gel samples were used, treated. and evaluated in the same way.

### 5.5. Statistics

Statistical analysis was performed using IBM SPSS Statistics software (version 25.0) and R software (version 4.1.2). Given the small sample size of observations, nonparametric descriptive statistics were used to describe the parameters collected during the study. For interval variables, the median (Me), the first and third quartiles (Q1; Q3), and the minimum and maximum were calculated. The results of the descriptive statistics of the interval variables are presented as Me [Q1; Q3]. Comparison of groups by interval variables was performed using the Wilcoxon–Mann–Whitney unpaired rank nonparametric test; for intragroup analysis, the Wilcoxon paired rank method or Friedman’s rank method was used. Differences were considered statistically significant at a significance level less than the designated alpha value, calculated taking into account the Bonferroni correction for multiple comparisons (for those cases where three pairs of comparisons were used, the calculated value of alpha = 0.05/3 = 0.0166; in those cases where seven pairs of comparisons were used, the calculated value of alpha = 0.05/7 = 0.0071). In the text of the manuscript, the significance levels are indicated either as absolute values or (in the case of exponential values) as *p* < 0.0001.

## Data Availability

Not applicable.

## Supplementary Materials

The following supporting information can be downloaded at: https://www.mdpi.com/article/10.3390/gels9090756/s1, Figure S1: FT-IR spectrum of N-(2-carboxyethyl)chitosan.
