# Supplementary material for: A Carboxyethylchitosan Gel Cross-Linked with Glutaraldehyde as a Candidate Carrier for Biomedical Applications
Source: Gels. 2023 Sep 16;9(9):756. doi: 10.3390/gels9090756 (PMC10531016; doi:10.3390/gels9090756)
Supplement: Supplementary file 1 [file gels-09-00756-s001.zip › gels-2508770-supplementary.pdf]

gels

Supplementary Materials:A Carboxyethylchitosan Gel Cross-Linked with Glutaraldehyde as a Candidate Carrier for Biomedical Applications

Anastasia Korel [1], Alexander Samokhin [1,*], Ekaterina Zemlyakova [2], Alexander Pestov [2], Elena Blinova [1], Maxim Zelikman [3], Vadim Tkachenko [4], Viktoria Bets [1], Svetlana Kretien [1,5], Elena Arzhanova [1] and Ekaterina Litvinova [1]

[1] Novosibirsk State Technical University, Faculty of physical engineering, 630073 Novosibirsk, Russia; akorel@gmail.com (A.K.); blinovaelena-85@yandex.ru (E.B.); vish22@yandex.ru (V.B.); ssonovo64@inbox.ru (S. K.); e.arzhanova@g.nsu.ru (E.A.); dimkit@mail.ru (E.L.)
[2] Institute of Organic Synthesis n.a. I. Ya. Postovsky UB RAS, 620137 Ekaterinburg, Russia; kottazem@mail.ru (E.Z.); pestov@ios.uran.ru (A.P.)
[3] Institute of Solid State Chemistry and Mechanochemistry SB RAS, 630090 Novosibirsk, Russia; zelikman_mv@mail.ru
[4] Institute of Nuclear Physics SB RAS, 630090 Novosibirsk, Russia; vtkachen@mail.ru
[5] Novosibirsk Research Institute of Traumatology and Orthopedics, 630091 Novosibirsk, Russia
* Correspondence: motorist@inbox.ru

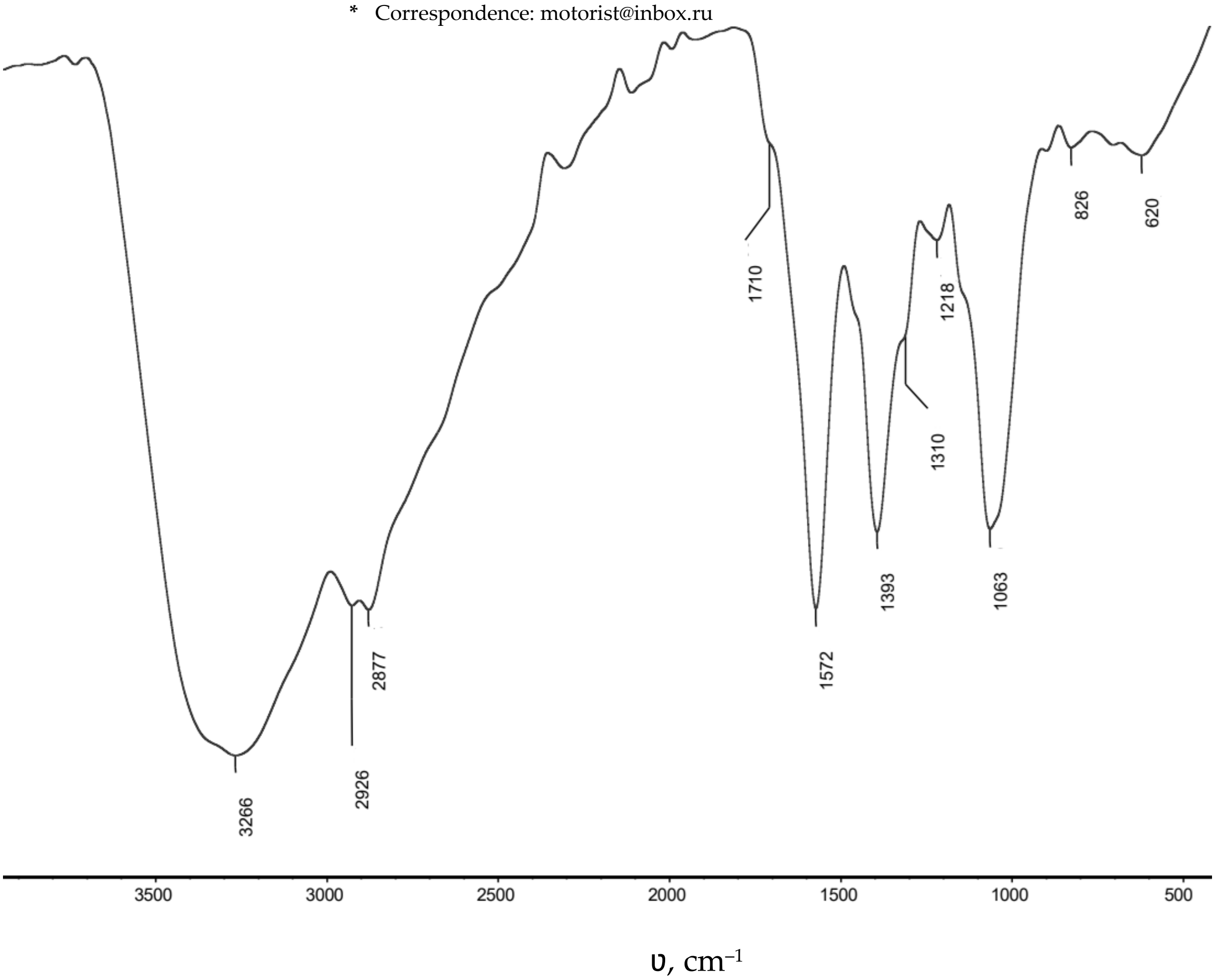

Figure S1.. FT-IR spectrum of N-(2-carboxyethyl)chitosan.
